# S33. ETRO - A PROSPECTIVE FOLLOW-UP STUDY OF THE COMBINED TREATMENT APPROACH “ROBIN” FOR ADOLESCENTS WITH HIGH RISK FOR DEVELOPING A PSYCHOTIC DISORDER: THERAPY MODULES ENHANCED BY A SMARTPHONE APPLICATION

**DOI:** 10.1093/schbul/sby018.820

**Published:** 2018-04-01

**Authors:** Maurizia Franscini, Nina Traber-Walker, Miriam Gerstenberg, Sibylle Metzler, Susanne Walitza

**Affiliations:** Psychiatric University Hospital Zurich

## Abstract

**Background:**

The most promising strategy in targeted prevention of psychotic disorders is to treat the at risk-symptoms in the pre-psychotic period. Although high risk-symptoms for psychotic disorder are common in adolescence and associated with a marked reduction in functioning, the evidence base required to guide effective interventions for adolescents at risk and even ﬁrst-episode psychosis is limited. The clinicians from the early intervention center in Zurich have developed the treatment approach “Robin” (standardized manual and smartphone App) for adolescents with high risk for developing a psychotic disorder. The manual is targeting at risk-symptoms, comorbid disorders, improvement of quality of life and daily functioning. The therapy modules are based on evidence based treatment strategies in adults with a high risk and recommendations for adolescents with first episodes of psychosis. It follows the guidelines on early intervention in clinical psychosis high risk states of the European Association for Psychiatry. The intervention also includes a smartphone application for supporting the patients between sessions. This application targets real-time symptom assessment, medication adherence, and provides coping strategies for dealing with symptoms of psychosis and daily life hurdles.

**Methods:**

The clinical intervention study ETRo is designed as a naturalistic controlled trial. The goal is to compare efficacy of a 16-week intervention in patients with at-risk symptoms (age range 13–18) with an active control group (treatment as usual). Power calculations conducted in collaboration with a statistician determined the recruitment goal of 30 participants in the treatment condition. Participants from a former early recognition study (N=62, Age: 13–18 years, Ø 15.06) are included for the control condition. For the intervention condition, help seeking adolescents with APS-Symptoms, aged 13–18, are being recruited during a three year time period. Within this prospective study, at-risk symptoms and data for comorbid symptoms, functioning, self-efficacy, and quality of life are collected at six time points (baseline, during the treatment period, immediately after intervention and 6, 12 and 24 months later).

**Results:**

Since August 2017, first participants have been included and their treatment has started. In Florence, we will present our first results. This will include implementation of the treatment program and first findings of treatment period.

**Discussion:**

Even though young patients with at-risk symptoms may profit best of specialised treatment approaches, little is known about age-appropriate treatment strategies in this vulnerable age group. This is one of the first controlled trials to test the efficacy of a specific treatment program for minor patients with attenuated psychotic symptoms.

